# Herpes simplex virus encephalitis: An atypical presentation of delirium

**DOI:** 10.1002/ccr3.7123

**Published:** 2023-03-26

**Authors:** Nicholas Wan, Sanath Weerakkody

**Affiliations:** ^1^ Department of General Medicine, Flinders Medical Centre Bedford Park South Australia Australia; ^2^ Adelaide Medical School University of Adelaide Adelaide South Australia Australia; ^3^ College of Medicine and Public Health, Flinders University Bedford Park South Australia Australia

**Keywords:** acute medicine, encephalitis, general medicine, herpes simplex virus, infectious diseases

## Abstract

Herpes simplex encephalitis (HSE) is the most common cause of infectious encephalitis. Our case is of a 75‐year‐old woman who presented with dysuria and altered mental status. Our case addresses the difficulties in diagnosis and highlights the importance of early recognition of HSE and its associated neurological sequelae.

## Graphical abstract

Herpes simplex encephalitis (HSE) is the most common cause of infectious encephalitis worldwide. Clinical features include fever and altered mental status—symptoms that are extremely common in clinical practice and nonspecific. It is important for the clinician to recognize the signs and commence empirical antiviral agents if HSE is suspected.

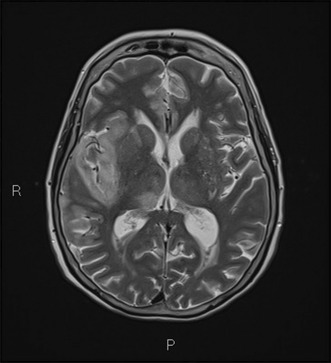

## 1 CASE PRESENTATION

A 75‐year‐old woman presented with a 3‐day history of fluctuating confusion, dysuria, and associated poor oral intake. Her past medical history includes previous ischemic heart disease and recurrent urinary tract infections. Prior to this, she had no recent hospital admissions and was otherwise eating and drinking well with no associated malaise or nausea. History taking was challenging as the patient was responsive but disoriented to time and place—throughout her history, she was frequently referring to her previous medical problems that had either resolved or were chronic in nature.

General inspection demonstrated a pale‐looking elderly female with a smaller‐than‐average body build. She was febrile at 38.1 degrees, with her other vital signs being within normal limits—blood pressure 111/65 mmhg, pulse rate 67 bpm, and spO2 96% on room air. On examination, her heart sounds were dual with no added sounds. She had good inspiratory effort with a clear chest with no associated cervical lymphadenopathy. Her abdomen was soft with mild tenderness in the right lower quadrant and suprapubic regions.

## 2 INVESTIGATIONS

Laboratory values demonstrated a white cell count of 11.32 × 10^9/L with her urine dipstick positive for nitrites and leukocytes. Initial urine cultures grew Escherichia coli sensitive to amoxicillin after 24 h of growth, with blood cultures growing the same pathogen soon after. She was commenced on intravenous amoxicillin and gentamycin for presumed urosepsis. She became afebrile after 24 h but remained persistently confused.

## 3 DIFFERENTIAL DIAGNOSIS

At this point, the main differential was delirium in the setting of known urosepsis. Her urine and blood grew Escherichia coli sensitive to amoxicillin, and she was on the appropriate antibiotics.

## 4 PROGRESS

**FIGURE 1:**
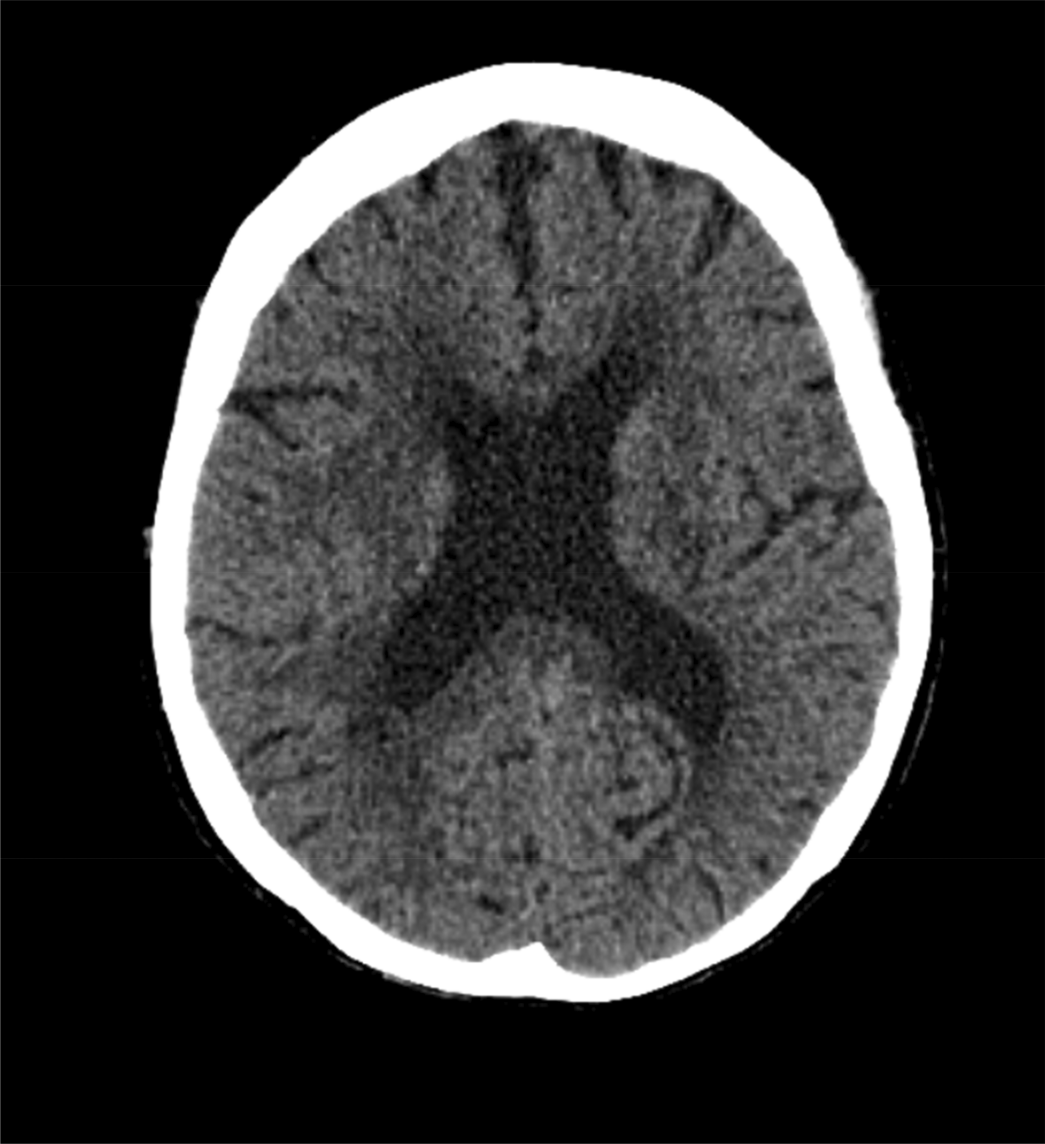
Initial computed tomography (CT) without contrast demonstrating no acute intracranial abnormality or hemorrhage or extra‐axial collections.

Forty‐eight hours later, she had a Glasgow Coma Scale (GCS) of 12 with a new oxygen requirement. A repeat septic screen demonstrated an increased focal air opacity in her left lung base demonstrating a new pneumonia and she was commenced on intravenous piperacillin‐tazobactam and azithromycin. Computed tomography of her brain demonstrated no acute intracranial pathology to explain her ongoing confusion (Figure 1). The main differential in this setting was a superimposed chest infection in an elderly patient who was delirious with urosepsis—of which she was already receiving escalated antibiotics.

Overnight, her GCS dropped further to three, and she developed full body tonic‐clonic seizures. She was febrile at 38.6 degrees with a left facial droop and a positive Babinski reflex. Her blood glucose was 6.3 mmol/L. A medical emergency team (MET) call was activated, resulting in the patient getting stabilized and intubated. The patient was treated with intravenous midazolam, levetiracetam, as well as antimicrobials including ceftriaxone and acyclovir.

**FIGURE 2:**
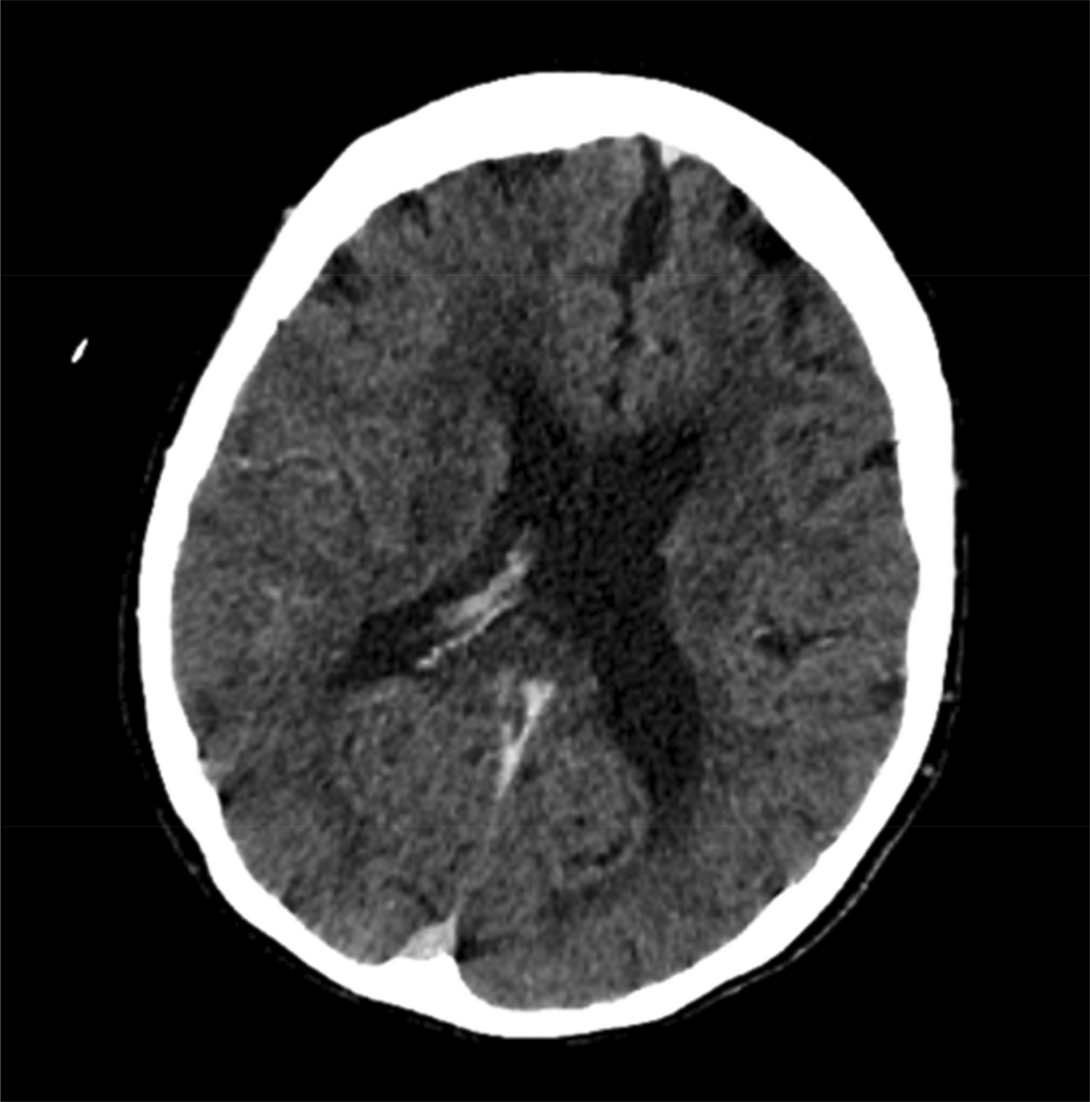
Repeat computed tomography (CT) with contrast demonstrating hypoattenuation in the right temporal lobe with loss of cortical medullary differentiation in the region of the right sylvian fissure, suspicious for an infective encephalitis.

Repeat computed tomography (CT) of her brain with contrast overnight revealed hypoattenuation in the right temporal lobe with loss of cortical medullary differentiation in the region of the right sylvian fissure, suspicious for an infective encephalitis (Figure 2).

**FIGURE 3:**
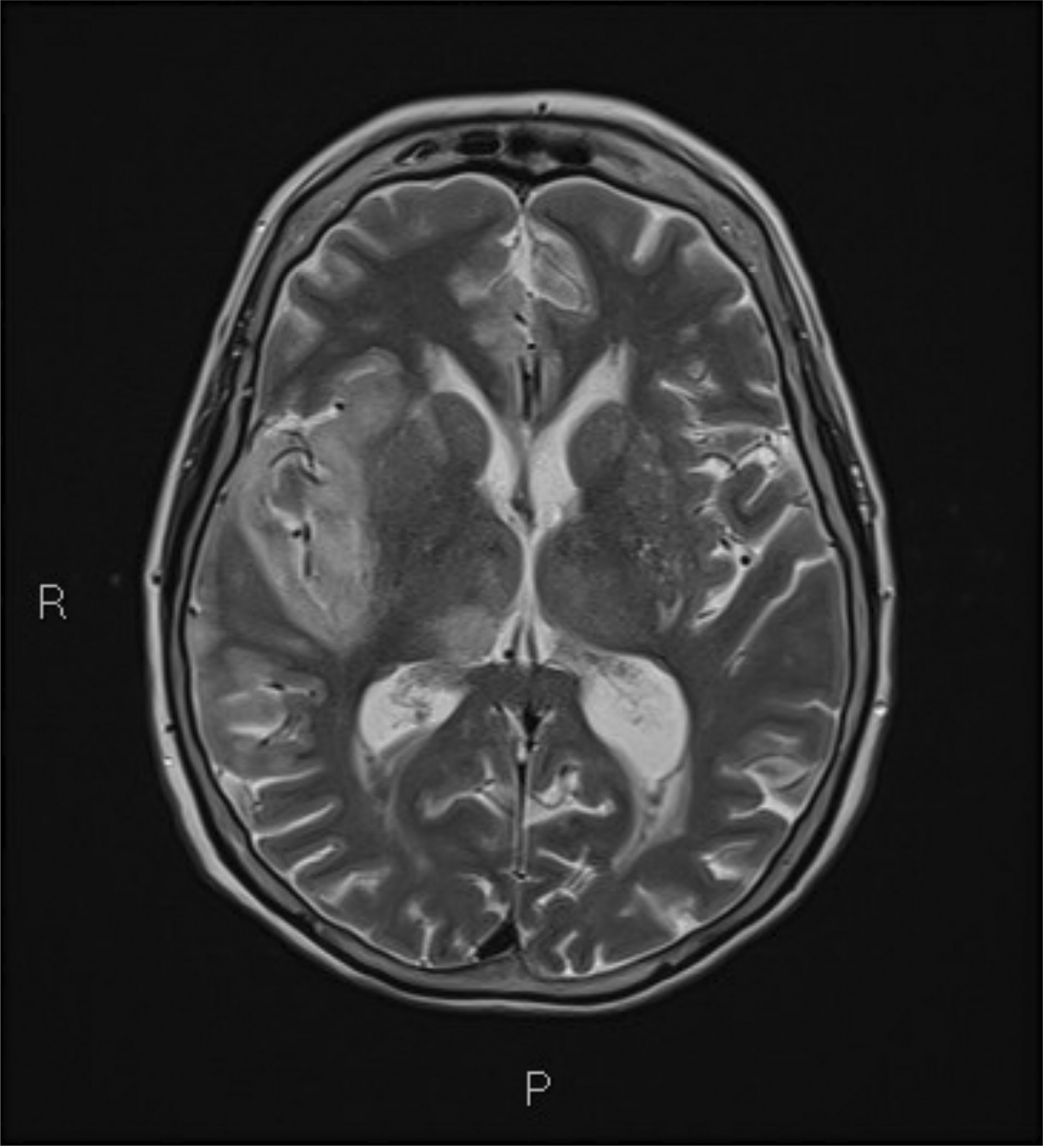
Magnetic resonance imaging (MRI) demonstrating generalized oedamatous changes of the right temporal lobe with moderate intensity restricted diffusion seen involving the gyri of the right temporal lobe and right insular regions.

Generalized edematous changes of the right temporal lobe was then seen on urgent magnetic resonance imaging (MRI), favoring the presumed diagnosis to be an infective encephalitis (Figure 3). Cerebral spinal fluid (CSF) samples from a lumbar puncture revealed monocytosis and tested positive for herpes simplex virus (HSV) on polymerase chain reaction (PCR).

## 5 OUTCOME AND FOLLOW‐UP

The patient was transferred to the intensive care unit (ICU) and was treated with intravenous acyclovir and antiepileptic medications. After 1 week of treatment, she was eventually made palliative as requested by her family due to unlikely favorable neurological recovery, most probably secondary to the delay in starting the specific treatment.

## 6 DISCUSSION

Herpes simplex virus encephalitis (HSE) is the most common cause of infectious encephalitis worldwide. It is estimated that HSV accounts for 10%–15% of cases of encephalitis annually.^1^ It involves a bimodal distribution, with one‐third of cases occurring in patients below 20 years of age while another half of cases occurring after the fifth decade of life.^2^ Both HSV‐1 and HSV‐2 are capable of causing disease but the HSV‐1 virus remains as the main pathogen involved in adults and also the predominant pathogen seen in neonates. It is estimated that up to 90% of the cases of HSE are caused by HSV‐1.^3^, ^4^

**FIGURE 4:**
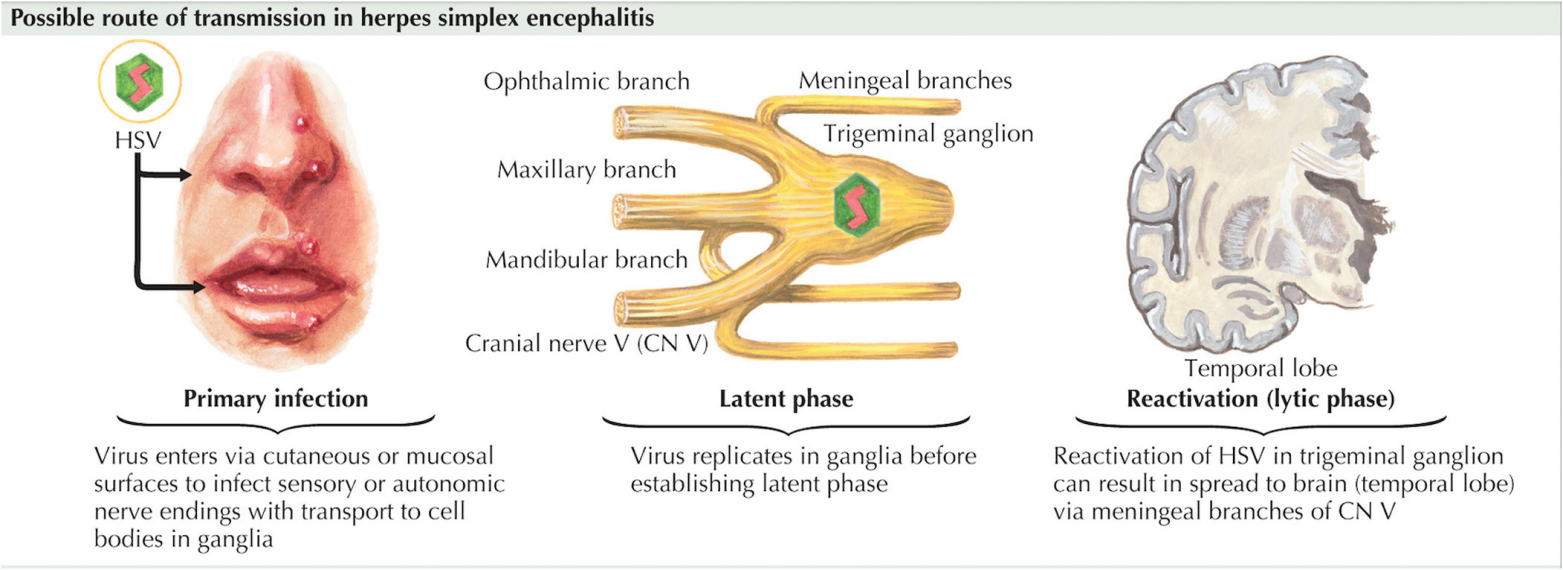
Proposed mechanisms and route of transmission in herpes simplex encephalitis (HSE).

While there have been several hypotheses proposed on the pathogenesis of HSE, its true pathophysiology remains incompletely understood. It is estimated that slightly less than a third of all HSE cases involve primary HSV infection while the remainder of the cases are attributed to latent HSV reactivation. Some of the proposed routes of infection involve:

1. Primary HSV‐1 infection of the oropharynx with trigeminal or olfactory involvement. In vivo experimentation on animal models have demonstrated the possibility of viral access via the trigeminal or olfactory nerves.^5^
2. Retrograde activation of latent HSV‐1 virus found in cell bodies in the trigeminal ganglion. This involves prior primary infection of HSV‐1, of what classically is described as “cold sores”.^6^ Figure 4 illustrates the aforementioned possible routes of transmission in HSE (Figure 4).^7^
3. CNS infection of HSV‐1 without any trigeminal involvement. This potentially represents reactivation of latent HSV‐1 virus found in cell bodies within the CNS. There have been several case studies suggesting the likelihood of HSV‐1 having the ability to remain latent in the CNS.^8^

Some of the classic histopathological features of HSE include necrotizing encephalitis involving the temporal lobe, frontal lobe, as well as the insular regions of the cerebral cortexes.^9^ As it is a hemorrhagic encephalitis, eosinophilic, or basophilic intranuclear inclusions composed of nucleic acid and protein, known eponymously as Cowdry bodies Type A, are usually seen on autopsy under light microscopy.^10^

Some of the clinical features of HSE include fever, altered mental status, focal seizures and headaches, focal seizures, ataxia, and dysphagia.^1^ Prodromal symptoms like behavioral changes and hallucinations might also be present. Symptoms usually gradually progress over a week but occasionally the time course can potentially be more rapid. Focal neurological deficits or altered sensorium with reversed sleep cycle can become clinically prominent. Table 1 presents a table of the percentage of patients demonstrating signs and symptoms of 388 reported HSE cases compiled by Gnann Jr and Whitley.^11^

**TABLE 1:** Percentage of patients demonstrating signs and symptoms of 388 reported HSE cases compiled by Gnann Jr and Whitley.

Finding	Percentage of patients	Reported range
Fever	80%	70%–97%
Confusion/Disorientation	72%	54%–81%
Personality changes/behavioral disturbances	59%	42%–92%
Headache	58%	42%–70%
Altered Mental Status/Impaired consciousness	58%	54%–100%
Seizures	54%	35%–65%
Focal Neurological Deficits	41%	26%–79%
Nausea and vomiting	40%	19%–46%
Aphasia/Altered Speech	40%	12%–65%
Coma	33%	4%–48%
Meningismus	28%	13%–38%

Magnetic resonance imaging with or without contrast is the imaging modality of choice for diagnosis, typically demonstrating asymmetric hyperintense lesions on T2‐weighted sequences.^12^ HSE is usually basal ganglia sparing on MRI, enabling the clinician to distinguish between HSE and middle cerebral artery infarcts.^13^

Lumbar puncture followed by CSF analysis and PCR remains the gold standard for establishing the diagnosis of HSE. It is estimated that CSF analysis and PCR has a sensitivity of 98% and a specificity of 94%–99%.^14^ Lymphocytic pleocytosis is usually seen, with up to 84 percent of patients demonstrating erythrocytosis.^15^

Electroencephalography (EEG) is not routinely performed in patients with HSE given the clinical time course; however, up to 80% of patients have abnormal EEG findings. Focal findings with prominent intermittent high amplitude slow waves (delta and theta slowing) are seen in up to 80% on patients with HSE. Classic findings of HSE on EEG include continuous periodic lateralized epileptiform discharges (PLED) in the affected region.^16^

If HSE is suspected, prompt empirical treatment of acyclovir is indicated.^6^ Aciclovir should be commenced intravenously at 10 mg/kg every 8 h for a minimum of 10–14 days.^6^ While it is tolerated at high doses, patients should be continued to be wellhydrated to avoid potential drug‐induced nephrotoxicity.^17^ It is essential to note that if the clinical picture is convincing for HSE, imaging studies and lumbar puncture should not delay the administration of treatment. As acyclovir is an antiviral agent working primarily by halting viral replication, it is crucial to commence treatment as soon as possible to prevent extensive replication and subsequent damage.

If prompt treatment is not administered, HSE has a high mortality rate of 70%.^2^ Mortality rates of patients receiving acyclovir is still at 20%, with up to 62% of survivors developing some form of neurologic sequalae.^6^ Relapses of HSE is extremely uncommon; however, they were seen in up to 12% of patients in one study, with majority of episodes occurring within 6 months after the initial disease.^18^ One post‐HSE complication includes the development of autoimmune NMDA encephalitis. It is thought that post‐HSE, IgG antibodies to the NR1 subunit of the NMDA receptor develop and affect its neuronal function. It has been reported in up to 27% in patients who previously had HSE.^19^

This case highlights the importance of recognizing HSE as one of the potential causes of fever and altered mental status. In the setting of a normal CT brain initially, the shrewd clinician should have a low threshold to consider alternative diagnoses. Given that this patient had three ongoing infections, it was unfortunate that prompt antiviral therapy was not given in time.

Delirium secondary to infectious etiologies is common; however, it is essential for the clinician to recognize early features of HSE and to always include HSE in the differential diagnosis of every febrile patient with an altered mental status. To prevent high mortality and morbidity as a result of delayed treatment, it is crucial to start specific empirical treatment at the earliest clinical suspicion.

## 7 LEARNING POINTS

Herpes simplex virus encephalitis is the most common cause of sporadic infective encephalitis worldwide, clinical features of HSV encephalitis include fever, altered mental status, headaches, focal seizures, dysphagia, and ataxia

1. Prompt recognition of symptoms and early empirical treatment of acyclovir 10 mg/kg IV every 8 h for 10–14 days should be commenced if clinical suspicion is high.
2. Lumbar puncture with CSF analysis and PCR remains the gold standard of diagnosis, showing lymphocytic pleocytosis and erythrocytosis (seen in up to 84% of patients).
3. Magnetic resonance imaging is the imaging modality of choice, demonstrating asymmetric hyperintense lesions on T2‐weighted sequences corresponding to areas of oedema. Lesions are usually found in medial temporal and inferior‐lateral frontal lobes.
4. The mortality of untreated patients remains high at 70%, while those receiving prompt treatment still remain high at 20%. Severe disability is seen in 20% of patients, with up to 62% of patients developing some form of neurologic sequelae.

## AUTHOR CONTRIBUTIONS

**Nicholas Ming Hao Wan:** Conceptualization; investigation; methodology; visualization; writing – original draft; writing – review and editing. **Sanath Weerakkody:** Conceptualization; investigation; methodology; project administration; supervision; visualization; writing – original draft; writing – review and editing.

## Acknowledgments

Open access publishing facilitated by The University of Adelaide, as part of the Wiley – The University of Adelaide agreement via the Council of Australian University Librarians.

## CONFLICT OF INTEREST STATEMENT

The author(s) declare no conflict of interest.

## CONSENT

Written informed consent was obtained from the patient to publish this report in accordance with the journal's patient consent policy.

## Data Availability

The data that support the findings of this study are available for free with public access on Pubmed where majority of these references were taken from. These data were derived from the following resources available in the public domain: Whitley RJ, Soong SJ, Linneman C, Jr., Liu C, Pazin G, Alford CA. Herpes simplex encephalitis. Clinical Assessment. JAMA. 1982;247(3):317‐20.2. Tyler KL. Herpes simplex virus infections of the central nervous system: encephalitis and meningitis, including Mollaret's. Herpes. 2004;11 Suppl 2:57A‐64A.3. Aurelius E, Johansson B, Skoldenberg B, Forsgren M. Encephalitis in immunocompetent patients due to herpes simplex virus type 1 or 2 as determined by type‐specific polymerase chain reaction and antibody assays of cerebrospinal fluid. J Med Virol. 1993;39(3):179‐86.Hjalmarsson A, Blomqvist P, Skoldenberg B. Herpes simplex encephalitis in Sweden, 1990‐2001: incidence, morbidity, and mortality. Clin Infect Dis. 2007;45(7):875‐80;Jennische E, Eriksson CE, Lange S, Trybala E, Bergstrom T. The anterior commissure is a pathway for contralateral spread of herpes simplex virus type 1 after olfactory tract infection. J Neurovirol. 2015;21(2):129‐47.6. Bradshaw MJ, Venkatesan A. Herpes Simplex Virus‐1 Encephalitis in Adults: Pathophysiology, Diagnosis, and Management. Neurotherapeutics. 2016;13(3):493‐508Runge MS. Herpes Simplex Encephalitis Netter's Internal Medicine 2009 8. Baringer JR, Pisani P. Herpes simplex virus genomes in human nervous system tissue analyzed by polymerase chain reaction. Ann Neurol. 1994;36(6):823‐9.9. Taylor SW, Smith RM, Pari G, Wobeser W, Rossiter JP, Jackson AC. Herpes simplex encephalitis. Can J Neurol Sci. 2005;32(2):246‐7.10. White CL, 3rd, Taxy JB. Early morphologic diagnosis of herpes simplex virus encephalitis: advantages of electron microscopy and immunoperoxidase staining. Hum Pathol. 1983;14(2):135‐9.11. Gnann JW, Jr., Whitley RJ. Herpes Simplex Encephalitis: an Update. Curr Infect Dis Rep. 2017;19(3):13.12. McCabe K, Tyler K, Tanabe J. Diffusion‐weighted MRI abnormalities as a clue to the diagnosis of herpes simplex encephalitis. Neurology. 2003;61(7):1015‐6.13. Domingues RB, Fink MC, Tsanaclis AM, de Castro CC, Cerri GG, Mayo MS, et al. Diagnosis of herpes simplex encephalitis by magnetic resonance imaging and polymerase chain reaction assay of cerebrospinal fluid. J Neurol Sci. 1998;157(2):148‐53.14. Binnicker MJ, Espy MJ, Irish CL. Rapid and direct detection of herpes simplex virus in cerebrospinal fluid by use of a commercial real‐time PCR assay. J Clin Microbiol. 2014;52(12):4361‐2.15. Nahmias AJ, Whitley RJ, Visintine AN, Takei Y, Alford CA, Jr. Herpes simplex virus encephalitis: laboratory evaluations and their diagnostic significance. J Infect Dis. 1982;145(6):829‐36.16. Kim YS, Jung KH, Lee ST, Kang BS, Yeom JS, Moon J, et al. Prognostic Value of Initial Standard EEG and MRI in Patients with Herpes Simplex Encephalitis. J Clin Neurol. 2016;12(2):224‐9.17. Pouplin T, Pouplin JN, Van Toi P, Lindegardh N, Rogier van Doorn H, Hien TT, et al. Valacyclovir for herpes simplex encephalitis. Antimicrob Agents Chemother. 2011;55(7):3624‐6.18. Skoldenberg B, Aurelius E, Hjalmarsson A, Sabri F, Forsgren M, Andersson B, et al. Incidence and pathogenesis of clinical relapse after herpes simplex encephalitis in adults. J Neurol. 2006;253(2):163‐70.19. Armangue T, Spatola M, Vlagea A, Mattozzi S, Carceles‐Cordon M, Martinez‐Heras E, et al. Frequency, symptoms, risk factors, and outcomes of autoimmune encephalitis after herpes simplex encephalitis: a prospective observational study and retrospective analysis. Lancet Neurol. 2018;17(9):760‐72.
